# Tetra­aqua­bis­(1,10-phenanthroline-κ^2^ *N*,*N*′)strontium 5,5′-diazene­diyl­ditetra­zolide

**DOI:** 10.1107/S1600536810039115

**Published:** 2010-10-09

**Authors:** Bao-Juan Jiao, Yi-Xia Ren, Gang Zhu, Zhi-Jun Yan, Fu-Rong Liu

**Affiliations:** a Department of Chemistry and Chemical Engineering, Xi’an University of Arts and Science, Xi’an 710065, Shaanxi, People’s Republic of China; b College of Chemistry and Chemical Engineering, Yanan University, Yanan 716000, Shaanxi, People’s Republic of China

## Abstract

The title complex, [Sr(C_12_H_8_N_2_)_2_(H_2_O)_4_](C_2_N_10_), contains an [Sr(phen)_2_(H_2_O)_4_]^2+^ cation (phen is 1,10-phenanthroline) and a 5,5′-diazenediylditetra­zolide anion (site symmetry 2). The Sr^2+^ cation (site symmetry 2) is coordinated by four N atoms from two chelating phen and four water mol­ecules. In the crystal structure, the water mol­ecules and the N atoms in the tetra­zolide rings form an extensive range of O—H⋯N hydrogen bonds which link the complex into a two-dimensional structure. An adjacent layer further yields a three-dimensional supramolecular network by offset face-to-face π–π stacking inter­actions of the phen ligands [with centroid–centroid distances of 3.915 (2) and 4.012 (2) Å]. The two bridging N atoms of the anion are equally disordered about the twofold rotation axis.

## Related literature

Tetra­zole compounds have been investigated as potential energy materials; see: Singh *et al.* (2006 ▶); Klapötke *et al.* (2009 ▶). In particular, complexes of tetra­zole containing cations such as strontium, barium or copper are components for pyrotechnical mixtures (Hartdegen *et al.*, 2009 ▶; Klapötke *et al.*, 2008 ▶). Additionally, the 5,5′-azotetra­zole with ten nitro­gen atoms is predicted to be involved in the hydrogen-bonding motif to construct a supra­molecule (Wang *et al.*, 2009 ▶). 
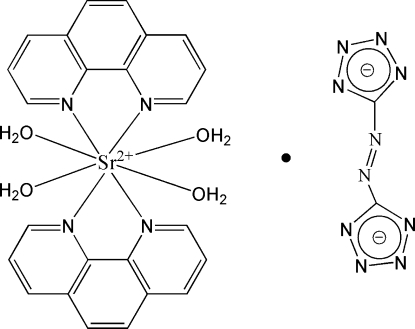


## Experimental

### 

#### Crystal data

[Sr(C_12_H_8_N_2_)_2_(H_2_O)_4_](C_2_N_10_)
*M* *_r_* = 684.21
Monoclinic, 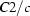

*a* = 17.442 (3) Å
*b* = 10.8974 (17) Å
*c* = 16.189 (3) Å
β = 105.178 (2)°
*V* = 2969.8 (8) Å^3^
*Z* = 4
Mo *K*α radiation
μ = 1.88 mm^−1^
*T* = 296 K
0.25 × 0.20 × 0.18 mm

#### Data collection

Bruker APEXII CCD diffractometer
Absorption correction: empirical (using intensity measurements) (*SADABS*; Bruker, 2002 ▶) *T*_min_ = 0.652, *T*_max_ = 0.729
7165 measured reflections
2621 independent reflections
2226 reflections with *I* > 2σ(*I*)
*R*_int_ = 0.026

#### Refinement

*R*[*F* ^2^ > 2σ(*F* ^2^)] = 0.027
*wR*(*F* ^2^) = 0.069
*S* = 1.04
2621 reflections
204 parameters
H-atom parameters constrained
Δρ_max_ = 0.31 e Å^−3^
Δρ_min_ = −0.37 e Å^−3^

### 

Data collection: *APEX2* (Bruker, 2007 ▶); cell refinement: *SAINT* (Bruker, 2007 ▶); data reduction: *SAINT*; program(s) used to solve structure: *SHELXS97* (Sheldrick, 2008 ▶); program(s) used to refine structure: *SHELXL97* (Sheldrick, 2008 ▶); molecular graphics: *SHELXTL* (Sheldrick, 2008 ▶); software used to prepare material for publication: *SHELXTL*.

## Supplementary Material

Crystal structure: contains datablocks I, global. DOI: 10.1107/S1600536810039115/jh2212sup1.cif

Structure factors: contains datablocks I. DOI: 10.1107/S1600536810039115/jh2212Isup2.hkl

Additional supplementary materials:  crystallographic information; 3D view; checkCIF report

## Figures and Tables

**Table 1:** Hydrogen-bond geometry (Å, °)

*D*—H⋯*A*	*D*—H	H⋯*A*	*D*⋯*A*	*D*—H⋯*A*
O2—H2*B*⋯N5^i^	0.85	2.08	2.885 (3)	158
O1—H1*A*⋯N4^i^	0.85	2.04	2.870 (2)	167
O2—H2*A*⋯N6^ii^	0.85	2.03	2.871 (3)	173
O1—H1*B*⋯N3	0.85	2.04	2.887 (3)	172

Symmetry codes: (i) 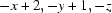; (ii) 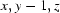.

**Fig. 1.**
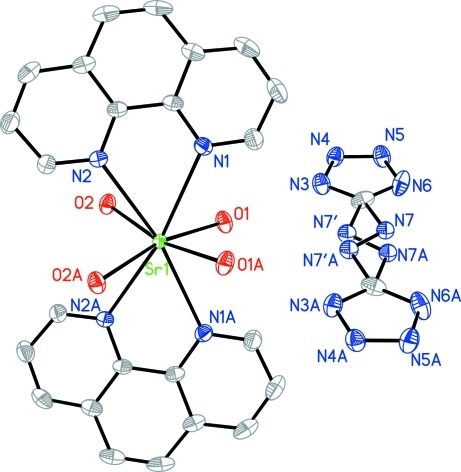
View of (I), A view of structure (I) showing the atom-numbering scheme. Displacement ellipsoids are drawn at the 30% probability level and hydrogen atoms are omitted for clarity. The occupancies of N7, are equal to 0.5 [Symmtry codes: A 2 - x,y,1/2 - z].

**Fig. 2.**
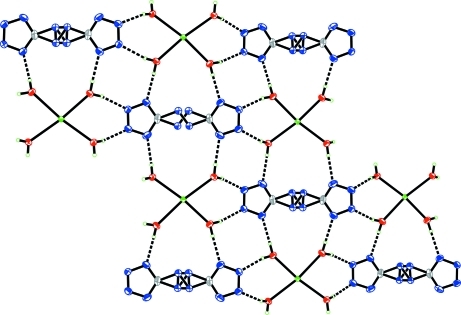
View of the supramolecular layer structure of (I) formed by the hydrogen bonds. The dashed lines are hydrogen bonds. Displacement ellipsoids are drawn at the 30% probability. The phen molecules are omitted for clarity.

## supplementary crystallographic information

### Comment

The high nitrogen content of tetrazole has led to investigation for their use as potential energy materials (Singh *et al.*, 2006; Klapötke *et al.*, 2009). Especially, complex of tetrazole containing cations like strontium, barium, or copper are sought components for pyrotechnical mixtures, by combination of the ligand and the colorantmetal cation (Hartdegen *et al.*, 2009; Klapötke *et al.*, 2008). Additionally, the 5,5'-azotetrazole with ten nitrogen atoms are predicted to be involved in the hydrogen bonds motif to construct supramolecule (Wang *et al.*, 2009). Herein, we report the crystal structure of the title compound, [Sr(phen)_2_(H_2_O)_4_][AT] (I), where phen = 1,10-phenanthroline and AT = 5,5'-diazenediylditetrazolide. The crystal structure of (I) consists of a discrete [Sr(phen)_2_(H_2_O)_4_]^2+^ cation and one 5,5'-diazenediylditetrazolide anion. As illustrated in Figure 1, the Sr^2+^ ion is coordinated by eight atoms with four N atoms from two phen molecules and four O atoms from water molecules, giving to a quadrangular prism structure. The N7 atom in the 5,5'-diazenediylditetrazolide anion is positional disordered and the occupancy of N7 must be set to 0.5 to get rational structure model and thermal displacement parameters. Strong hydrogen bonds between the 5,5'-diazenediylditetrazolide anion and water molecules link neighboring [Sr(phen)_2_(H_2_O)_4_]^2+^ cations, which giving to a two dimensional supramolecular layer, as shown in the Figure 2. Furthermore, the adjacent layers were form to a three dimensional supramolecular network, by the off-set face to face π-π stacking interactions of the phen molecules, with the centroid distance 3.915 and 4.012 Å.

### Experimental

30 ml H_2_O containing 2.0 mmol (0.6003 g) disodium 5,5'-azotetrazole pentahydrate was mixed with 30 ml e thanol containing 4.0 mmol (0.7929 g) 1,10-phenanthroline. 15 ml H_2_O containing 2.0 mmol (0.5332 g) SrCl_2_.6H_2_O was added to the above mixture. Yellow single crystals were obtained from the mixture solution which was allowed to evaporate at the room temperature for two weeks.

### Refinement

The H atoms of C atoms were positioned geometrically and refined with a riding model, with C—H = 0.93 Å and Å and *U*_iso_(H) = 1.2*U*_eq_(C).The water H atoms were located in difference Fourier maps,with distance restraints of O—H = 0.85±0.02 Å, and then refined with isotropic thermal parameters 1.5 times those of O atoms.

### Crystal data

**Table:**

[Sr(C_12_H_8_N_2_)_2_(H_2_O)_4_](C_2_N_10_)	*F*(000) = 1392
*M**_r_* = 684.21	*D*_x_ = 1.530 Mg m^−^^3^
Monoclinic, *C*2/*c*	Mo *K*α radiation, λ = 0.71073 Å
*a* = 17.442 (3) Å	Cell parameters from 3217 reflections
*b* = 10.8974 (17) Å	θ = 2.2–26.9°
*c* = 16.189 (3) Å	µ = 1.88 mm^−^^1^
β = 105.178 (2)°	*T* = 296 K
*V* = 2969.8 (8) Å^3^	Block, yellow
*Z* = 4	0.25 × 0.20 × 0.18 mm

### Data collection

**Table:**

Bruker APEXII CCD diffractometer	2621 independent reflections
Radiation source: fine-focus sealed tube	2226 reflections with *I* > 2σ(*I*)
graphite	*R*_int_ = 0.026
φ and ω scans	θ_max_ = 25.0°, θ_min_ = 2.2°
Absorption correction: empirical (using intensity measurements) (*SADABS*; Bruker, 2002)	*h* = −20→20
*T*_min_ = 0.652, *T*_max_ = 0.729	*k* = −12→11
7165 measured reflections	*l* = −19→16

### Refinement

**Table:**

Refinement on *F*^2^	Secondary atom site location: difference Fourier map
Least-squares matrix: full	Hydrogen site location: inferred from neighbouring sites
*R*[*F*^2^ > 2σ(*F*^2^)] = 0.027	H-atom parameters constrained
*wR*(*F*^2^) = 0.069	*w* = 1/[σ^2^(*F*_o_^2^) + (0.0334*P*)^2^ + 1.6965*P*] where *P* = (*F*_o_^2^ + 2*F*_c_^2^)/3
*S* = 1.04	(Δ/σ)_max_ < 0.001
2621 reflections	Δρ_max_ = 0.31 e Å^−^^3^
204 parameters	Δρ_min_ = −0.37 e Å^−^^3^
0 restraints	Extinction correction: *SHELXL97* (Sheldrick, 2008), Fc^*^=kFc[1+0.001xFc^2^λ^3^/sin(2θ)]^-1/4^
Primary atom site location: structure-invariant direct methods	Extinction coefficient: 0.0049 (3)

### Special details

**Table:**

Geometry. All e.s.d.'s (except the e.s.d. in the dihedral angle between two l.s. planes) are estimated using the full covariance matrix. The cell e.s.d.'s are taken into account individually in the estimation of e.s.d.'s in distances, angles and torsion angles; correlations between e.s.d.'s in cell parameters are only used when they are defined by crystal symmetry. An approximate (isotropic) treatment of cell e.s.d.'s is used for estimating e.s.d.'s involving l.s. planes.
Refinement. Refinement of *F*^2^ against ALL reflections. The weighted *R*-factor *wR* and goodness of fit *S* are based on *F*^2^, conventional *R*-factors *R* are based on *F*, with *F* set to zero for negative *F*^2^. The threshold expression of *F*^2^ > σ(*F*^2^) is used only for calculating *R*-factors(gt) *etc*. and is not relevant to the choice of reflections for refinement. *R*-factors based on *F*^2^ are statistically about twice as large as those based on *F*, and *R*- factors based on ALL data will be even larger.

### Fractional atomic coordinates and isotropic or equivalent isotropic displacement parameters (Å^2^)

**Table:**

	*x*	*y*	*z*	*U*_iso_*/*U*_eq_	Occ. (<1)
Sr1	1.0000	0.24389 (2)	0.2500	0.02718 (12)	
N1	0.85008 (10)	0.34918 (17)	0.18410 (12)	0.0395 (4)	
N2	0.85933 (10)	0.10403 (17)	0.21752 (11)	0.0372 (4)	
N3	0.96163 (12)	0.64644 (18)	0.09038 (13)	0.0483 (5)	
N4	0.94140 (12)	0.68916 (18)	0.01083 (12)	0.0443 (5)	
N5	0.93980 (13)	0.80929 (19)	0.01267 (13)	0.0499 (5)	
N6	0.95898 (14)	0.8474 (2)	0.09331 (15)	0.0580 (6)	
C1	0.84222 (15)	0.4689 (2)	0.17143 (17)	0.0515 (6)	
H1	0.8839	0.5192	0.1998	0.062*	
C2	0.77523 (17)	0.5244 (3)	0.11807 (19)	0.0643 (8)	
H2	0.7729	0.6091	0.1110	0.077*	
C3	0.71337 (16)	0.4521 (3)	0.07654 (18)	0.0640 (8)	
H3	0.6685	0.4872	0.0401	0.077*	
C4	0.71751 (14)	0.3253 (3)	0.08878 (15)	0.0477 (6)	
C5	0.65475 (15)	0.2429 (3)	0.04826 (18)	0.0628 (8)	
H5	0.6094	0.2741	0.0102	0.075*	
C6	0.65995 (15)	0.1226 (3)	0.06392 (17)	0.0609 (8)	
H6	0.6183	0.0716	0.0365	0.073*	
C7	0.72830 (13)	0.0706 (2)	0.12204 (15)	0.0461 (6)	
C8	0.73506 (15)	−0.0545 (2)	0.14216 (17)	0.0551 (7)	
H8	0.6943	−0.1082	0.1165	0.066*	
C9	0.80122 (15)	−0.0976 (2)	0.19914 (17)	0.0534 (7)	
H9	0.8061	−0.1802	0.2139	0.064*	
C10	0.86160 (14)	−0.0145 (2)	0.23494 (16)	0.0457 (6)	
H10	0.9067	−0.0448	0.2739	0.055*	
C11	0.78741 (12)	0.2779 (2)	0.14439 (14)	0.0374 (5)	
C12	0.79253 (12)	0.1478 (2)	0.16157 (13)	0.0356 (5)	
C13	0.97136 (14)	0.7456 (2)	0.13849 (15)	0.0476 (6)	
O1	1.01607 (10)	0.39982 (14)	0.13806 (9)	0.0463 (4)	
O2	0.98909 (9)	0.10539 (13)	0.11891 (9)	0.0422 (4)	
H1A	1.0306	0.3855	0.0928	0.063*	
H2B	1.0196	0.1165	0.0863	0.063*	
H2A	0.9765	0.0299	0.1132	0.063*	
H1B	0.9960	0.4708	0.1253	0.063*	
N7	0.9905 (2)	0.7903 (3)	0.2248 (2)	0.0372 (9)*	0.50
N7'	0.9955 (3)	0.6972 (3)	0.2281 (2)	0.0402 (10)*	0.50

### Atomic displacement parameters (Å^2^)

**Table:**

	*U*^11^	*U*^22^	*U*^33^	*U*^12^	*U*^13^	*U*^23^
Sr1	0.02894 (17)	0.02498 (17)	0.02556 (16)	0.000	0.00346 (10)	0.000
N1	0.0351 (10)	0.0383 (11)	0.0444 (11)	0.0036 (8)	0.0091 (8)	0.0031 (8)
N2	0.0303 (9)	0.0381 (11)	0.0410 (10)	−0.0018 (8)	0.0055 (8)	−0.0004 (8)
N3	0.0600 (13)	0.0401 (12)	0.0475 (12)	0.0021 (10)	0.0188 (10)	0.0097 (10)
N4	0.0579 (13)	0.0402 (12)	0.0357 (11)	−0.0054 (10)	0.0137 (9)	−0.0052 (9)
N5	0.0661 (14)	0.0409 (12)	0.0468 (13)	0.0056 (10)	0.0219 (11)	0.0081 (10)
N6	0.0777 (16)	0.0429 (13)	0.0628 (15)	−0.0153 (11)	0.0351 (12)	−0.0181 (11)
C1	0.0492 (14)	0.0412 (15)	0.0646 (17)	0.0034 (12)	0.0158 (13)	0.0056 (12)
C2	0.0677 (19)	0.0477 (16)	0.080 (2)	0.0209 (15)	0.0230 (16)	0.0199 (15)
C3	0.0494 (16)	0.076 (2)	0.0629 (18)	0.0234 (15)	0.0091 (14)	0.0244 (15)
C4	0.0358 (13)	0.0629 (17)	0.0431 (14)	0.0105 (12)	0.0079 (11)	0.0109 (12)
C5	0.0306 (13)	0.100 (3)	0.0496 (16)	0.0040 (15)	−0.0043 (11)	0.0083 (15)
C6	0.0347 (14)	0.085 (2)	0.0545 (16)	−0.0105 (14)	−0.0030 (12)	−0.0043 (15)
C7	0.0343 (12)	0.0602 (16)	0.0429 (14)	−0.0072 (11)	0.0084 (10)	−0.0061 (12)
C8	0.0468 (15)	0.0569 (17)	0.0618 (17)	−0.0206 (13)	0.0145 (13)	−0.0149 (13)
C9	0.0529 (15)	0.0402 (14)	0.0688 (17)	−0.0090 (12)	0.0190 (13)	−0.0052 (12)
C10	0.0404 (13)	0.0387 (14)	0.0560 (15)	−0.0007 (10)	0.0089 (11)	−0.0003 (11)
C11	0.0276 (11)	0.0495 (14)	0.0352 (12)	0.0036 (10)	0.0085 (9)	0.0029 (10)
C12	0.0274 (11)	0.0474 (13)	0.0321 (11)	−0.0010 (10)	0.0077 (9)	−0.0024 (10)
C13	0.0404 (13)	0.0722 (19)	0.0315 (12)	−0.0124 (12)	0.0114 (10)	−0.0074 (13)
O1	0.0655 (11)	0.0362 (9)	0.0407 (9)	0.0076 (8)	0.0203 (8)	0.0097 (7)
O2	0.0585 (10)	0.0339 (8)	0.0353 (8)	−0.0051 (7)	0.0142 (7)	−0.0054 (6)

### Geometric parameters (Å, °)

**Table:**

Sr1—O1^i^	2.5527 (15)	C4—C5	1.435 (4)
Sr1—O1	2.5527 (15)	C5—C6	1.334 (4)
Sr1—O2	2.5704 (14)	C5—H5	0.9300
Sr1—O2^i^	2.5704 (14)	C6—C7	1.429 (3)
Sr1—N1^i^	2.7985 (18)	C6—H6	0.9300
Sr1—N1	2.7985 (18)	C7—C8	1.400 (4)
Sr1—N2	2.8185 (17)	C7—C12	1.413 (3)
Sr1—N2^i^	2.8185 (17)	C8—C9	1.358 (4)
N1—C1	1.322 (3)	C8—H8	0.9300
N1—C11	1.358 (3)	C9—C10	1.394 (3)
N2—C10	1.321 (3)	C9—H9	0.9300
N2—C12	1.361 (3)	C10—H10	0.9300
N3—C13	1.317 (3)	C11—C12	1.442 (3)
N3—N4	1.327 (3)	C13—N7	1.434 (4)
N4—N5	1.310 (3)	C13—N7'	1.496 (4)
N5—N6	1.327 (3)	O1—H1A	0.8503
N6—C13	1.314 (3)	O1—H1B	0.8520
C1—C2	1.396 (3)	O2—H2B	0.8499
C1—H1	0.9300	O2—H2A	0.8500
C2—C3	1.363 (4)	N7—N7^i^	0.797 (6)
C2—H2	0.9300	N7—N7'	1.018 (5)
C3—C4	1.395 (4)	N7—N7'^i^	1.254 (5)
C3—H3	0.9300	N7'—N7'^i^	0.686 (6)
C4—C11	1.410 (3)	N7'—N7^i^	1.254 (5)
			
O1^i^—Sr1—O1	96.53 (7)	C11—C4—C5	119.4 (2)
O1^i^—Sr1—O2	168.21 (5)	C6—C5—C4	121.5 (2)
O1—Sr1—O2	78.63 (5)	C6—C5—H5	119.3
O1^i^—Sr1—O2^i^	78.63 (5)	C4—C5—H5	119.3
O1—Sr1—O2^i^	168.21 (5)	C5—C6—C7	121.3 (2)
O2—Sr1—O2^i^	108.08 (7)	C5—C6—H6	119.4
O1^i^—Sr1—N1^i^	73.85 (5)	C7—C6—H6	119.4
O1—Sr1—N1^i^	74.48 (5)	C8—C7—C12	117.8 (2)
O2—Sr1—N1^i^	114.54 (5)	C8—C7—C6	123.0 (2)
O2^i^—Sr1—N1^i^	93.80 (5)	C12—C7—C6	119.2 (2)
O1^i^—Sr1—N1	74.48 (5)	C9—C8—C7	119.9 (2)
O1—Sr1—N1	73.85 (5)	C9—C8—H8	120.0
O2—Sr1—N1	93.80 (5)	C7—C8—H8	120.0
O2^i^—Sr1—N1	114.54 (5)	C8—C9—C10	118.2 (2)
N1^i^—Sr1—N1	131.59 (8)	C8—C9—H9	120.9
O1^i^—Sr1—N2	103.91 (5)	C10—C9—H9	120.9
O1—Sr1—N2	118.66 (5)	N2—C10—C9	124.7 (2)
O2—Sr1—N2	69.86 (5)	N2—C10—H10	117.7
O2^i^—Sr1—N2	73.09 (5)	C9—C10—H10	117.7
N1^i^—Sr1—N2	166.84 (5)	N1—C11—C4	123.1 (2)
N1—Sr1—N2	57.97 (5)	N1—C11—C12	118.00 (18)
O1^i^—Sr1—N2^i^	118.66 (5)	C4—C11—C12	118.9 (2)
O1—Sr1—N2^i^	103.91 (5)	N2—C12—C7	122.1 (2)
O2—Sr1—N2^i^	73.09 (5)	N2—C12—C11	118.15 (19)
O2^i^—Sr1—N2^i^	69.86 (5)	C7—C12—C11	119.7 (2)
N1^i^—Sr1—N2^i^	57.97 (5)	N6—C13—N3	112.7 (2)
N1—Sr1—N2^i^	166.84 (5)	N6—C13—N7	102.6 (2)
N2—Sr1—N2^i^	114.53 (7)	N3—C13—N7	144.7 (3)
C1—N1—C11	117.0 (2)	N6—C13—N7'	143.2 (3)
C1—N1—Sr1	120.81 (16)	N3—C13—N7'	104.1 (2)
C11—N1—Sr1	120.12 (14)	N7—C13—N7'	40.60 (19)
C10—N2—C12	117.23 (19)	Sr1—O1—H1A	127.1
C10—N2—Sr1	120.88 (14)	Sr1—O1—H1B	131.5
C12—N2—Sr1	119.09 (14)	H1A—O1—H1B	98.8
C13—N3—N4	104.26 (19)	Sr1—O2—H2B	120.3
N5—N4—N3	109.29 (18)	Sr1—O2—H2A	128.2
N4—N5—N6	109.49 (18)	H2B—O2—H2A	104.9
C13—N6—N5	104.26 (19)	N7^i^—N7—N7'	86.5 (3)
N1—C1—C2	124.0 (2)	N7^i^—N7—N7'^i^	54.1 (2)
N1—C1—H1	118.0	N7'—N7—N7'^i^	33.1 (3)
C2—C1—H1	118.0	N7^i^—N7—C13	157.9 (4)
C3—C2—C1	118.8 (3)	N7'—N7—C13	73.0 (3)
C3—C2—H2	120.6	N7'^i^—N7—C13	106.1 (3)
C1—C2—H2	120.6	N7'^i^—N7'—N7	92.7 (3)
C2—C3—C4	119.8 (2)	N7'^i^—N7'—N7^i^	54.2 (2)
C2—C3—H3	120.1	N7—N7'—N7^i^	39.4 (3)
C4—C3—H3	120.1	N7'^i^—N7'—C13	159.0 (2)
C3—C4—C11	117.2 (2)	N7—N7'—C13	66.4 (3)
C3—C4—C5	123.4 (2)	N7^i^—N7'—C13	105.4 (3)
			
O1^i^—Sr1—N1—C1	58.23 (18)	C1—N1—C11—C4	2.7 (3)
O1—Sr1—N1—C1	−43.51 (18)	Sr1—N1—C11—C4	−160.92 (17)
O2—Sr1—N1—C1	−120.51 (18)	C1—N1—C11—C12	−176.1 (2)
O2^i^—Sr1—N1—C1	127.64 (18)	Sr1—N1—C11—C12	20.3 (3)
N1^i^—Sr1—N1—C1	7.47 (17)	C3—C4—C11—N1	−1.4 (4)
N2—Sr1—N1—C1	176.1 (2)	C5—C4—C11—N1	178.8 (2)
N2^i^—Sr1—N1—C1	−125.5 (3)	C3—C4—C11—C12	177.3 (2)
O1^i^—Sr1—N1—C11	−138.79 (17)	C5—C4—C11—C12	−2.4 (3)
O1—Sr1—N1—C11	119.47 (17)	C10—N2—C12—C7	−1.1 (3)
O2—Sr1—N1—C11	42.47 (16)	Sr1—N2—C12—C7	160.13 (16)
O2^i^—Sr1—N1—C11	−69.38 (17)	C10—N2—C12—C11	177.5 (2)
N1^i^—Sr1—N1—C11	170.45 (17)	Sr1—N2—C12—C11	−21.3 (2)
N2—Sr1—N1—C11	−20.89 (15)	C8—C7—C12—N2	0.0 (3)
N2^i^—Sr1—N1—C11	37.5 (3)	C6—C7—C12—N2	−180.0 (2)
O1^i^—Sr1—N2—C10	−117.05 (17)	C8—C7—C12—C11	−178.5 (2)
O1—Sr1—N2—C10	137.34 (17)	C6—C7—C12—C11	1.5 (3)
O2—Sr1—N2—C10	73.43 (17)	N1—C11—C12—N2	0.9 (3)
O2^i^—Sr1—N2—C10	−43.77 (17)	C4—C11—C12—N2	−177.9 (2)
N1^i^—Sr1—N2—C10	−38.6 (3)	N1—C11—C12—C7	179.5 (2)
N1—Sr1—N2—C10	−178.36 (19)	C4—C11—C12—C7	0.7 (3)
N2^i^—Sr1—N2—C10	13.94 (16)	N5—N6—C13—N3	0.6 (3)
O1^i^—Sr1—N2—C12	82.42 (15)	N5—N6—C13—N7	−179.0 (2)
O1—Sr1—N2—C12	−23.18 (17)	N5—N6—C13—N7'	178.2 (4)
O2—Sr1—N2—C12	−87.10 (15)	N4—N3—C13—N6	−0.5 (3)
O2^i^—Sr1—N2—C12	155.71 (16)	N4—N3—C13—N7	178.8 (4)
N1^i^—Sr1—N2—C12	160.9 (2)	N4—N3—C13—N7'	−179.1 (2)
N1—Sr1—N2—C12	21.11 (14)	N6—C13—N7—N7^i^	−154.7 (17)
N2^i^—Sr1—N2—C12	−146.58 (16)	N3—C13—N7—N7^i^	26 (2)
C13—N3—N4—N5	0.3 (3)	N7'—C13—N7—N7^i^	22.7 (16)
N3—N4—N5—N6	0.1 (2)	N6—C13—N7—N7'	−177.5 (4)
N4—N5—N6—C13	−0.4 (3)	N3—C13—N7—N7'	3.2 (6)
C11—N1—C1—C2	−2.2 (4)	N6—C13—N7—N7'^i^	−178.9 (3)
Sr1—N1—C1—C2	161.3 (2)	N3—C13—N7—N7'^i^	1.7 (6)
N1—C1—C2—C3	0.5 (4)	N7'—C13—N7—N7'^i^	−1.4 (5)
C1—C2—C3—C4	0.8 (4)	N7^i^—N7—N7'—N7'^i^	10.9 (10)
C2—C3—C4—C11	−0.4 (4)	C13—N7—N7'—N7'^i^	−177.5 (8)
C2—C3—C4—C5	179.3 (3)	N7'^i^—N7—N7'—N7^i^	−10.9 (10)
C3—C4—C5—C6	−177.7 (3)	C13—N7—N7'—N7^i^	171.6 (7)
C11—C4—C5—C6	2.0 (4)	N7^i^—N7—N7'—C13	−171.6 (7)
C4—C5—C6—C7	0.2 (5)	N7'^i^—N7—N7'—C13	177.5 (8)
C5—C6—C7—C8	178.0 (3)	N6—C13—N7'—N7'^i^	11 (3)
C5—C6—C7—C12	−1.9 (4)	N3—C13—N7'—N7'^i^	−171 (2)
C12—C7—C8—C9	1.1 (4)	N7—C13—N7'—N7'^i^	7(2)
C6—C7—C8—C9	−178.9 (3)	N6—C13—N7'—N7	4.1 (6)
C7—C8—C9—C10	−1.2 (4)	N3—C13—N7'—N7	−178.1 (4)
C12—N2—C10—C9	1.1 (4)	N6—C13—N7'—N7^i^	−1.3 (6)
Sr1—N2—C10—C9	−159.8 (2)	N3—C13—N7'—N7^i^	176.4 (3)
C8—C9—C10—N2	0.0 (4)	N7—C13—N7'—N7^i^	−5.5 (4)

Symmetry codes: (i) −*x*+2, *y*, −*z*+1/2.

### Hydrogen-bond geometry (Å, °)

**Table:**

*D*—H···*A*	*D*—H	H···*A*	*D*···*A*	*D*—H···*A*
O2—H2B···N5^ii^	0.85	2.08	2.885 (3)	158
O1—H1A···N4^ii^	0.85	2.04	2.870 (2)	167
O2—H2A···N6^iii^	0.85	2.03	2.871 (3)	173
O1—H1B···N3	0.85	2.04	2.887 (3)	172

Symmetry codes: (ii) −*x*+2, −*y*+1, −*z*; (iii) *x*, *y*−1, *z*.

## References

[bb1] Bruker (2002). *SADABS* Bruker AXS Inc., Madison, Wisconsin, USA.

[bb2] Bruker (2007). *APEX2* and *SAINT* Bruker AXS Inc., Madison, Wisconsin, USA.

[bb3] Hartdegen, V., Klapötke, T. M. & Sproll, S. M. (2009). *Inorg. Chem.* **48**, 9549–9556. 10.1021/ic901413n 19780625

[bb4] Klapötke, T. M., Sabate, C. M. & Stierstorfer, J. (2008). *Z. Anorg. Allg. Chem.* **634**, 1867–1874.

[bb5] Klapötke, T. M., Sabate, C. M. & Welch, J. M. (2009). *Eur. J. Inorg. Chem.* **2009**, 769–776.

[bb6] Sheldrick, G. M. (2008). *Acta Cryst.* A**64**, 112–122. 10.1107/S0108767307043930 18156677

[bb7] Singh, R. P., Verma, R. D., Meshri, D. T. & Shreeve, J. M. (2006). *Angew. Chem. Int. Ed.* **45**, 3584–3601. 10.1002/anie.200504236 16708411

[bb8] Wang, W. T., Chen, S. P., Fan, G., Xie, G., Jiao, B. & Gao, S. (2009). *J. Coord. Chem.* **62**, 1879–1886.

